# Circulating fibrocytes traffic to the lung in murine acute lung injury and predict outcomes in human acute respiratory distress syndrome: a pilot study

**DOI:** 10.1186/s10020-020-00176-0

**Published:** 2020-05-27

**Authors:** Christine M. Lin, Abdullah Alrbiaan, John Odackal, Zhimin Zhang, Yogesh Scindia, Sun-Sang J. Sung, Marie D. Burdick, Borna Mehrad

**Affiliations:** 1 Division of Pulmonary, Critical Care, and Sleep Medicine, University of Florida, 1600 SW Archer Road, Box 100225, Gainesville, FL 32610-0225 USA; 2 Department of Medicine, University of Virginia, Charlottesville, VA USA

**Keywords:** Acute lung injury, Respiratory distress syndrome (adult), Pneumonia, Fibrosis, Biomarkers

## Abstract

**Background:**

Fibrosis is an integral component of the pathogenesis of acute lung injury and is associated with poor outcomes in patients with acute respiratory distress syndrome (ARDS). Fibrocytes are bone marrow-derived cells that traffic to injured tissues and contribute to fibrosis; hence their concentration in the peripheral blood has the potential to serve as a biomarker of lung fibrogenesis. We therefore sought to test the hypothesis that the concentration and phenotype of circulating fibrocytes in patients with ARDS predicts clinical outcomes.

**Methods:**

For the animal studies, C57Bl/6 mice were infected with experimental *Klebsiella pneumoniae* in a model of acute lung injury; one-way ANOVA was used to compare multiple groups and two-way ANOVA was used to compare two groups over time. For the human study, 42 subjects with ARDS and 12 subjects with pneumonia (without ARDS) were compared to healthy controls. Chi-squared or Fisher’s exact test were used to compare binary outcomes. Survival data was expressed using a Kaplan-Meier curve and compared by log-rank test. Univariable and multivariable logistic regression were used to predict death.

**Results:**

In mice with acute lung injury caused by *Klebsiella* pneumonia, there was a time-dependent increase in lung soluble collagen that correlated with sequential expansion of fibrocytes in the bone marrow, blood, and then lung compartments. Correspondingly, when compared via cross-sectional analysis, the initial concentration of blood fibrocytes was elevated in human subjects with ARDS or pneumonia as compared to healthy controls. In addition, fibrocytes from subjects with ARDS displayed an activated phenotype and on serial measurements, exhibited intermittent episodes of markedly elevated concentration over a median of 1 week. A peak concentration of circulating fibrocytes above a threshold of > 4.8 × 10^6^ cells/mL cells correlated with mortality that was independent of age, ratio of arterial oxygen concentration to the fraction of inspired oxygen, and vasopressor requirement.

**Conclusions:**

Circulating fibrocytes increase in a murine model of acute lung injury and elevation in the number of these cells above a certain threshold is correlated with mortality in human ARDS. Therefore, these cells may provide a useful and easily measured biomarker to predict outcomes in these patients.

## Background

Acute respiratory distress syndrome (ARDS) remains among the most common complications of critical illness, resulting in 20–40% mortality and, amongst survivors, substantial long-term morbidity (Thompson et al. 2017). The identification of clinically relevant prognostic biomarkers in ARDS would constitute a significant advance in the field by identifying patients that may benefit from tailored interventions.

Fibroproliferation is a key feature of ARDS pathogenesis. While early autopsy studies divided the course of ARDS sequentially into an early exudative, subsequent proliferative, and late fibrotic phases (Katzenstein et al. 1976), contemporary understanding indicates that fibrogenesis can be detected as early as 24 h after the onset of ARDS (Armstrong et al. 1999; Marshall et al. 2000) and that the extent of fibrosis correlates with prolonged mechanical ventilation and death (Martin et al. 1995). Even in the era of low-tidal volume ventilation, survivors of ARDS demonstrate persistent evidence of lung fibrosis for months to years after the illness, which is associated with impaired lung function and health-related quality of life (Burnham et al. 2013; Burnham et al. 2014). Biomarkers of fibrogenesis in bronchoalveolar lavage fluid of ARDS patients have been shown to predict outcomes in several cross-sectional studies (Wang et al. 2019; Capelozzi et al. 2017), but these tests are limited by the need for bronchoscopy, and related to that, the impracticality of assessing biomarkers longitudinally. To date, few studies have examined the prognostic potential of peripheral blood biomarkers of fibrosis in ARDS, or made such measurements serially (Capelozzi et al. 2017; Terpstra et al. 2014).

Myofibroblasts are the principal effector cells that mediate tissue remodeling and fibroproliferation. In the context of the lung, potential sources of myofibroblasts include differentiation from resident lung mesenchymal cells, transformation of resident cells of non-mesenchymal lineages, and recruitment of bone marrow-derived circulating progenitor cells, known as fibrocytes. Fibrocytes are released from the bone marrow into the bloodstream and are recruited to injured tissues in the context of both physiologic wound healing and pathologic fibrosis (Keeley et al. 2009); as such, fibrocytes may represent a biomarker of tissue fibrogenesis that is easily measurable in the peripheral blood. Since fibrocytes have been shown to accumulate in the bronchoalveolar lavage fluid in patients with ARDS (Quesnel et al. 2012), we sought to test the hypothesis that the number or phenotype of circulating fibrocytes in patients with ARDS predicts clinical outcomes.

## Methods

### Animals and in vivo procedures

C57BL/6 J mice were purchased from the Jackson Laboratory (Bar Harbor, ME) and maintained under specific pathogen-free conditions. Experiments were performed in age- and sex-matched 6–12-week-old animals in compliance with Institutional Animal Care and Use Committee approved protocols. Experimental bacterial pneumonia was induced as previously described (Mehrad et al. 2006; Chen et al. 2013) by intra-tracheal inoculation of 500 colony-forming units of *Klebsiella pneumoniae* strain 43,816 (American Type Culture Collection, Manassas, VA), an inoculum that resulted in < 20% mortality between days 4–7 of infection. At designated time points, mice were euthanized with an overdose of ketamine and xylazine. Blood was collected from the right ventricle into heparinized syringes and the pulmonary vasculature was perfused with 2 ml of phosphate buffered saline (PBS) containing 2 mM EDTA via the right ventricle, before harvesting the lobes of the lungs and the left femur. Cell suspensions from the blood buffy coat, lung, and bone marrow were prepared as previously described (Bettina et al. 2016; Barletta et al. 2012). In some experiments, bone marrow was collected in PBS, cells disrupted with ultrasound, filtered to remove debris, and the resulting fluid concentrated to 100 μL with centrifugal filters with 3 kDa molecular weight cut-off (Amicon Ultra-4, Millipore-Sigma). Bronchoalveolar lavage was performed as described (Park et al. 2006). Mouse CXCL12 (Luminex, Austin, TX), albumin (Bethyl Laboratories, Montgomery, TX), and soluble collagen (Sircol collagen assay, Biocolor, Belfast, UK) were quantified per manufacturers’ instructions.

### Confocal microscopy

Confocal microscopic analysis of murine lungs was performed as described (Sung et al. 2006). Briefly, mice were euthanized with ketamine and xylazine and lungs were perfused with 20 mL cold PBS via the right ventricle. Lungs were fix-inflated with periodate-lysine- *p*-formaldehyde fixative (McLean and Nakane 1974) for 30 min at 20 cm hydrostatic pressure via a 22 G catheter ligated to the trachea. Lungs lobes were then resected and further fixed in periodate-lysine- *p*-formaldehyde for 2.5 h. After equilibration in 5% sucrose in 50 mM phosphate buffer (pH 7.4) overnight, 2 h in 15% sucrose, and 4 h in 30% sucrose, the tissues were embedded in Optimal Cutting Temperature compound. Sections (5 μm) for staining were extracted with 0.3% triton X-100, blocked with anti-FcγRII/FcγRIII mAb (clone 2.4G2) and serum, and stained with fluorochrome-linked antibodies. The following antibodies were used: A647-conjugated anti-CD45 (clone 30-F11; BioLegend, San Diego, CA); A700-anti-alpha smooth muscle actin (α-SMA) mAb (clone 1A4; R&D Systems, Minneapolis, MN), A555-rabbit anti-mouse N-terminal pro-collagen I (Cloud Clone, Houston, TX), and A488-goat anti-mouse collagen III (Southern Biotech, Birmingham, AL). Anti-pro-collagen I and anti-collagen III antibodies were conjugated with Alexa Fluor mAb labeling kits (Invitrogen, Grand Island, NY) before cell labeling. Confocal microscopy was performed on a Zeiss LSM700 assembly with 405, 488, 543, and 633 nm excitation lines. Data were compiled using ZEN software (Zeiss, Thornton, NY).

### Human samples

Subjects were enrolled under institutionally approved protocols and provided written informed consent. Inclusion criteria were admission to the intensive care unit and either ARDS, as defined by the Berlin definition (Ranieri et al. 2012) or pneumonia, defined as the presence of air space disease on chest imaging plus two of the following: fever (T > 38.5 °C), purulent sputum production, and peripheral white blood cell count > 10 or < 4 × 10^9^ cells/L. These diagnoses were independently ascertained by two investigators (BM and either CML or AA). Healthy controls were volunteers ≥18 years of age who were non-smokers and without acute or chronic medical illnesses. Exclusion criteria were pregnancy, surgery, or trauma within the previous month. ARDS and pneumonia subjects were recruited within 24 h after ICU admission, on whom venous blood samples were collected on the day of enrollment and every other day for up to 14 days, or until liberation from mechanical ventilation (in ARDS subjects) or transfer out of the ICU (in pneumonia subjects). Survival data was subsequently determined for each patient through query of the electronic medical record at up to 3 years after initial enrollment. Blood samples were drawn in 10 mL sodium heparin tubes and processed as described (Keeley et al. 2012; Trimble et al. 2014). Briefly, samples were transported on ice and refrigerated at 4 °C overnight, without ex vivo manipulations such as cell enrichment, freezing, or culture. In preliminary studies, blood fibrocyte count values analyzed immediately after blood draw and after overnight storage differed by < 5% (data not shown). Plasma was separated by centrifugation and stored at − 80 °C and peripheral blood buffy coats were prepared as previously described (Mehrad et al. 2017; Mehrad et al. 2007) and processed for flow cytometry. Human CXCL2, latent TGF-β, active TGF-β, C-terminal pro-peptide of collagen I, N-terminal pro-peptide of collagen I, and N-terminal pro-peptide of collagen III were quantified by ELISA per manufacturers’ instructions.

### Flow cytometry

Fibrocyte populations were quantified, as previously described, in human blood (Keeley et al. 2012; Trimble et al. 2014; Mehrad et al. 2017; Shipe et al. 2016; Shields et al. 2018) and murine bone marrow, blood, and lungs (Field et al. 2012; Mehrad et al. 2009). Briefly, red blood cells were lysed, samples were filtered through 100 μm nylon mesh, and live cells were enumerated under a hemocytometer by trypan blue exclusion. Cells were resuspended at 10^7^ cells per ml, and 10^6^ cells were incubated for 20 min at 4 °C with human IgG (Millipore-Sigma, Burlington, MA, USA) or anti-mouse anti-CD16/CD32 (clone 2.4G2; BD Biosciences, San Jose, CA, USA), and were then stained with isotype control or the following surface antibodies (from BD Biosciences except when stated otherwise): anti-human CD45-V500 (clone H130), anti-human CXCR4-APC (clone 12G5), anti-mouse CD45-PerCP (clone 30-F11), anti-mouse CCR2-APC (clone 475,301; R&D Systems, Minneapolis, MN, USA) anti-mouse CCR7 PE-Cy7 (clone 4B12; Thermo Fisher Scientific, Waltham, MA, USA) and anti-mouse CXCR4-Pacific Blue (clone 2B11). Cells were then permeabilized (Cytofix/Cytoperm kit, BD Biosciences) and stained with the following intracellular antibodies or isotype controls: anti-α-SMA-PE (R&D Systems), anti-collagen-1 (Col-1) DyLight 488 (Rockland, Gilbertsville, PA, USA), and anti-mouse decapentaplegic homolog-2 and -3 phosphorylated at serine 433 or 435 (p-SMAD-2/3; Santa Cruz Biotechnology, Santa Cruz, CA, USA). Anti-collagen and respective control antibodies were first conjugated to DyLight 488 using DyLight conjugation kit (Thermo Fisher Scientific) according to the manufacturer’s instructions. After staining, samples were fixed in 2% paraformaldehyde and data were acquired on a FACS Canto II flow cytometer using BD Diva software (BD Biosciences). Absolute concentration of fibrocytes and fibrocyte subsets was calculated as the proportion of the cell population, as determined by flow cytometry, multiplied by the concentration of nucleated cells in the original sample.

### Statistical analysis

Data were analyzed using SAS (version 9.4 for Windows, Cary, NC, USA) and Prism (version 8 for Mac, GraphPad, San Diego, CA, USA). Euler diagrams were generated with EulerAPE (version 3.0.0; open source, http://www.eulerdiagrams.org/eulerAPE/). Summary data were presented as the mean ± standard error of the mean (SEM) or median ± interquartile range (IQR). Kruskal-Wallis test was used to compare multiple groups, and two-way ANOVA to compare two groups over time. Chi-squared or Fisher’s exact test were used for comparison of binary outcomes between human subject groups. For prospective survival outcome stratification, the optimal threshold value of peak fibrocytes was determined by maximizing overall prediction performance with the Youden-*J* index from a receiver operating characteristic analysis. Association between peak fibrocyte count exceeding a threshold value and death was assessed using the Fisher’s exact test, and survival data was expressed using Kaplan-Meier curves and compared by log-rank test. Univariable and multivariable logistic regression were used to predict death. Two-sided probability values of less than 0.05 were considered statistically significant.

## Results

**Fig. 1:**
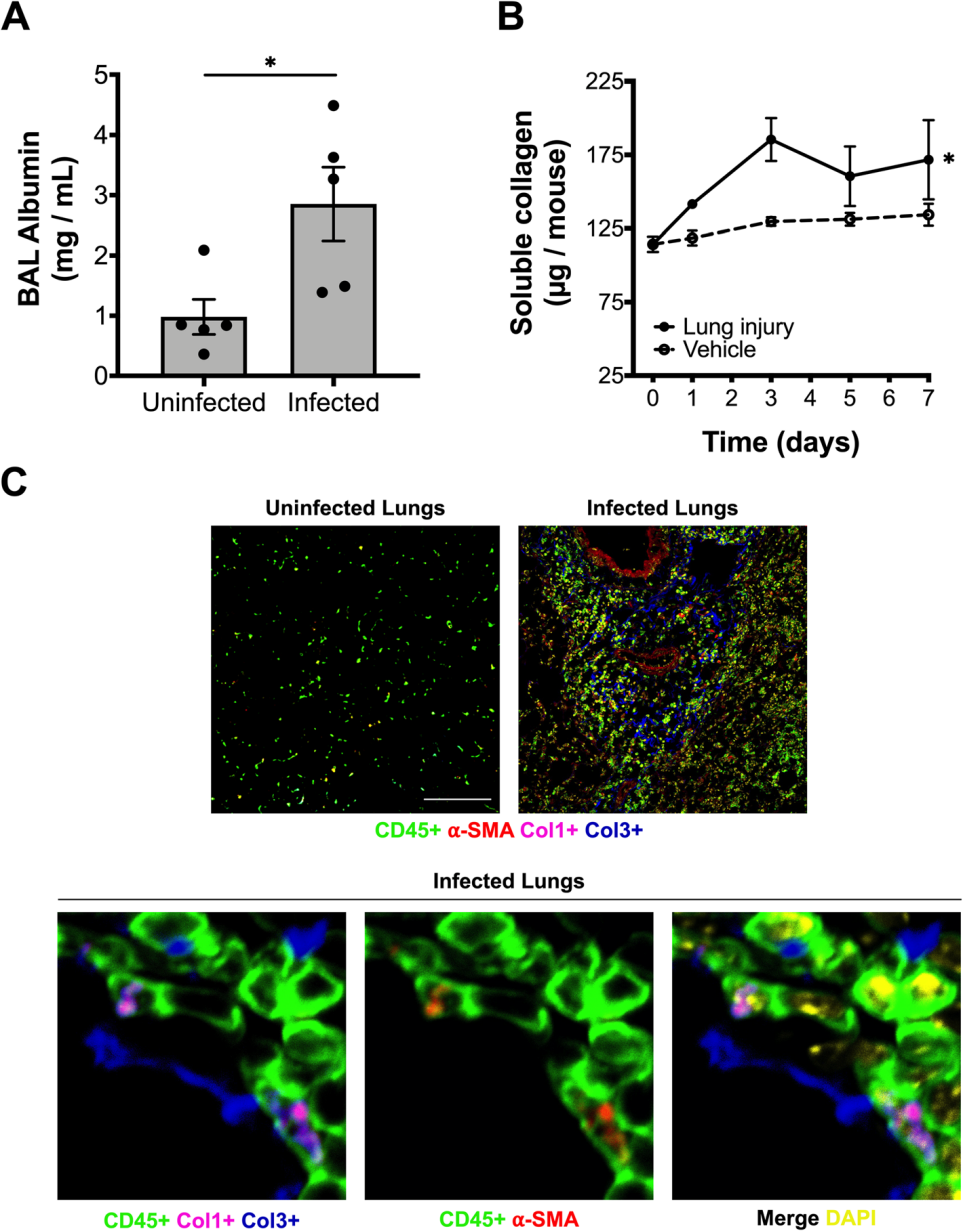
Gram-negative pneumonia results in murine acute lung injury with changes in lung collagen content and localization of collagen-producing cells. **a** Bronchoalveolar lavage (BAL) albumin concentration on day 3 of acute lung injury caused by experimental *Klebsiella* pneumonia. Each dot represents one animal; mean and SEM are indicated. **b** Change in lung soluble collagen after onset of acute lung injury; *n* = 4–6 experimental animals per time point per group. **c** Initial panel at low power magnification (scale bar = 50 μm) demonstrates lung immunofluorescent staining of normal (uninfected) lung tissue showing staining for CD45, N-terminal pro-collagen I (proCol1), collagen III (Col3), α-smooth muscle actin (α-SMA), and nuclear stain (DAPI). Subsequent panels show high-power magnification demonstrate representative images on day 3 of lung injury. *, *p* < 0.05 by Mann-Whitney test (panel A) and 2-way ANOVA (panel B)

**Fig. 2:**
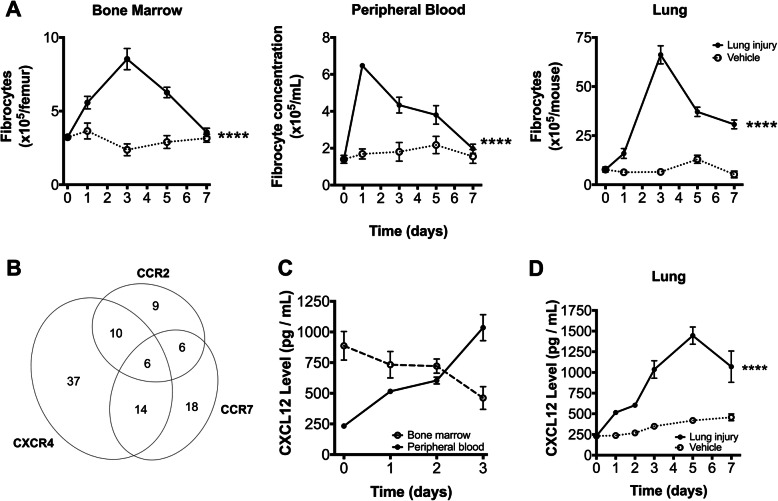
Fibrocytes in murine acute lung injury caused by Gram-negative pneumonia. **a** Concentration of fibrocytes in the bone marrow, blood, and lung compartments after onset of acute lung injury. **b** Distribution of median percentage of lung fibrocytes expressing chemokine receptors on day 3 acute lung injury. **c**-**d** Concentration of CXCL12 in the bone marrow, peripheral blood, and lung after onset of acute lung injury. Panels A, C, and D show data as mean ± SEM. All data is representative of 5–6 animals per group and per time point. *, *p* < 0.05; ****, *p* < 0.0001 by 2-way ANOVA (panels **a**, **d**) and Kruskal-Wallis test (panel **c**)

Since bacterial pneumonia is among the most common causes of ARDS (Moss and Mannino 2002), we began by evaluating fibrocytes in an established model of acute lung injury caused by multifocal *Klebsiella* pneumonia in mice*.* Infected mice experienced significant acute lung injury and disruption of the capillary-alveolar barrier as evidence by 3-fold increase in the concentration of bronchoalveolar lavage (BAL) albumin in comparison to control (Fig. 1a). To quantify the extent of associated fibrogenesis, we serially measured lung soluble collagen following the onset of infection. As compared to animals inoculated with saline vehicle, lung soluble collagen progressively increases as lung injury progressed in mice with pneumonia (Fig. 1b) in comparison to vehicle control (*p* = 0.04). Immunohistochemical staining showed infected lungs to contain CD45+ cells containing pro-collagen-I, collagen-III, and myofibroblast differentiation marker, α-SMA (Fig. 1c), consistent with an activated fibrocyte phenotype (Keeley et al. 2009). We then enumerated fibrocyte populations in the bone marrow, blood, and lung compartments over time. As compared to animals inoculated with saline vehicle, the population of fibrocytes increased markedly in all three compartments in infected animals but with different dynamics: peak fibrocyte concentration occurred on day 1 of infection in the blood and on day 3 of infection in the bone marrow and lung, consistent with rapid release of fibrocytes from the bone marrow into the blood, and slower recruitment from the blood to the lungs and expansion of fibrocytes in the bone marrow (Fig. 2a).

We subsequently assessed the expression of the chemokine receptors CXCR4, CCR2, and CCR7 on lung fibrocytes, since these receptors have been previously implicated in fibrocyte trafficking (Moore et al. 2005; Phillips et al. 2004; Sakai et al. 2006). In all compartments, CXCR4 was expressed by 67% of fibrocytes, either alone or in combination with other receptors; CCR2 and CCR7 were expressed by smaller subsets (Supplemental Fig. 1 and Fig. 2b). We next measured the levels of CXCL12, the ligand of CXCR4, in the bone marrow, blood, and lung compartments. Over the first 3 days of the infection, the concentration of CXCL12 in the bone marrow compartment halved, whereas the concentration in the peripheral blood and lungs increased 4-fold (Fig. 2c-d), consistent with a gradient of CXCL12 between the bone marrow and the lungs.

In order to assess the relevance of circulating fibrocytes as biomarkers in human ARDS, we enrolled 42 subjects with ARDS, 12 subjects with pneumonia without concurrent ARDS who had been admitted to an intensive care unit, and 20 healthy control subjects. The groups were demographically similar. As compared to subjects with pneumonia alone, those with ARDS had worse oxygenation and a significantly higher rate of mechanical ventilation, vasopressor requirement, and PEEP, but comparable modified SOFA scores, ICU length of stay, and mortality (Table 1).

**Table 1:** Characteristics of study subjects

**Demographic Information**	**ARDS** **(*n*** **= 42)**	**Pneumonia without ARDS** **(*n*** **= 12)**	**Healthy Controls** **(*n*** **= 20)**	***p*value**
**Age/years (median and IQR)**	54 (41–63)	56 (48–70)	55 (46–62)	0.51
**Sex (% male)**	57	42	50	0.61
**Race (%)**				
**Caucasian**	83	67	60	0.19
**African American**	17	33	35	
**Asian**	0	0	5	
**mSOFA score at enrollment (median and IQR)**	7 (6–9)	6 (3–9)	–	0.36
**Mechanical ventilation (%)**	98	33	–	< 0.0001
**Type (% conventional)**	100	100		0.99
**PEEP (median and IQR)**	12 (10–14)	8 (7–9)		0.01
**Vt (median and IQR)**	400 (350–448)	380 (345–400)		0.39
**P:F ratio at enrollment**	123 (99–156)	161 (156–193)	–	0.02
**Vasopressor use (%)**	67	25	–	0.01
**Immunocompromised (%)**	31	33	–	0.99
**Cause of ARDS (%)**			–	
**Pneumonia**	76	–	–	
**Sepsis**	10	–	–	
**Aspiration**	7	–	–	
**Other**	7	–	–	
**ICU length of stay/days (median and IQR)**	7 (5–10)	6 (1–12)	–	0.35
**In-hospital mortality (%)**	38	33	–	0.99

*Abbreviations*: ARDS, acute respiratory distress syndrome; mSOFA, modified sequential organ failure assessment score (excluding the Glasgow coma scale component); ICU, intensive care unit; IQR, inter-quartile range; PEEP, positive end-expiratory pressure, Vt, tidal volume (in mL), P:F ratio, ratio of PaO_2_ to FIO_2_. Types of mechanical ventilation within 24 h of enrollment included conventional (volume- or pressure-controlled mode, extra-corporeal membrane oxygenation (ECMO), or high frequency oscillatory ventilation (HFOV). “Other” causes of ARDS were two cases of acute pancreatitis and one case of catastrophic antiphospholipid antibody syndrome

**Fig. 3:**
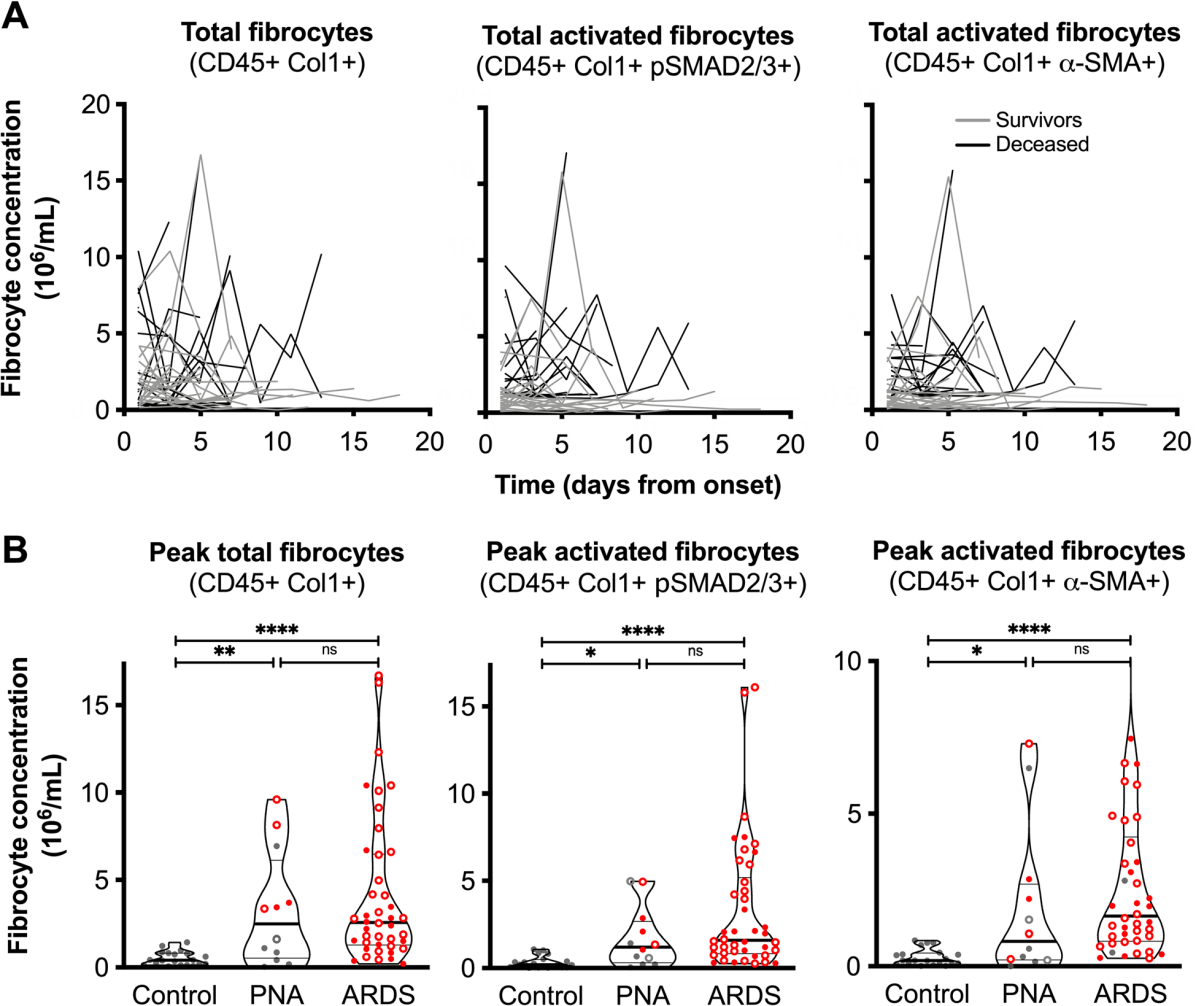
Circulating fibrocytes in human pneumonia and ARDS. **a** Change over time in the number of total fibrocytes (CD45+ Col1+) and activated fibrocytes staining for phosphorylated SMAD-2 or − 3 (CD45+ Col1+ pSMAD2/3+) or expressing alpha-smooth muscle actin (CD45+ Col1+ α-SMA+) during the course of ARDS. Each line represents one subject. **b** Comparison of peak total fibrocytes (CD45+ Col1+) and activated fibrocytes (CD45+ Col1+ pSMAD2/3+ and CD45+ Col1+ α-SMA+). Each dot represents one subject; bold horizontal lines indicate the median, and light horizontal lines represent the 25th and 75th percentiles. Red dots indicate patients that required mechanical ventilation; open dots indicate patients that required vasopressor support. *, *p* < 0.05; **, *p* < 0.01, ****, *p* < 0.0001 by Kruskal-Wallis test

When evaluated serially over the course of a subject’s ICU stay, we found the absolute concentration of circulating fibrocytes in ARDS patients to display intermittent episodes of marked elevation (Fig. 3a). To assess the activation state of these cells, we measured the subset of fibrocytes expressing α-SMA similar to measurements in murine lung injury. In addition, we quantified fibrocytes staining for the phosphorylated forms of SMAD-2 and -3, which identify cells with TGF-β receptor signaling. We found a similar pattern of fluctuation of concentrations of these activated fibrocyte subsets, indicating that the expanded circulating fibrocyte pool in patients with ARDS were activated (Fig. 3a). In cross-sectional comparisons, the median concentration of circulating fibrocytes was significantly higher in subjects with ARDS or pneumonia as compared to healthy controls, both on the first day of ICU admission (Supplemental Fig. 2), and, more markedly, when comparing the peak concentration of fibrocytes (Fig. 3b). Similar to the observations in the murine model, CXCR4 was the most commonly expressed chemokine receptor, followed by CCR7 and CCR2 (Supplemental Fig. 3). Taken together, these data suggest that acute lung injury, including in non-ARDS pneumonia, causes an elevation in circulating fibrocyte concentration in both mice and humans.

We next assessed the subjects with ARDS for other circulating biomarkers relevant to fibrogenesis, namely plasma CXCL12, latent and active TGF-β, and pro-peptides of collagen-I and -III. In contrast to fibrocytes, none of these variables differed significantly between healthy subjects, subjects with pneumonia, and those with ARDS, with values of many subjects being below the level of detection (Supplemental Fig. 4). In addition, none of these variables correlated significantly with circulating fibrocyte concentrations (data not shown).

**Fig. 4:**
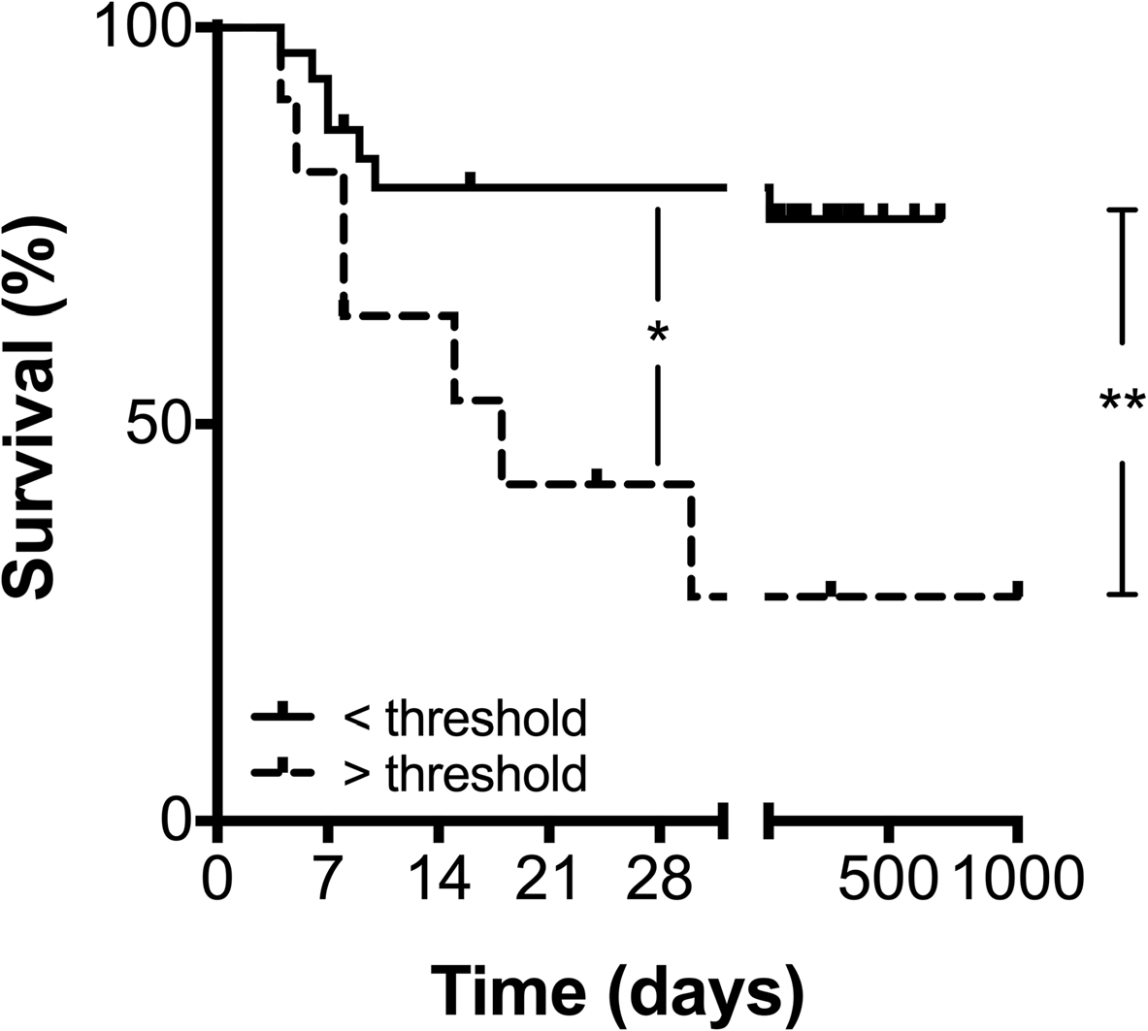
Circulating fibrocyte concentration as predictors of mortality in subjects with ARDS. Kaplan-Meier survival analysis of subjects with ARDS, separated by peak circulating activated fibrocyte concentration threshold value of 4.8 × 10^6^ cells/mL. Of the 42 subjects with ARDS, 9 subjects had values above and 33 subjects had values below the threshold. *, *p* = 0.028 at day 28 and **, *p =* 0.009 for the entire follow-up period by Log-rank test

Lastly, we sought to identify a threshold fibrocyte concentration that would identify the risk of death in subjects with ARDS. A peak circulating fibrocyte concentration of > 4.8 × 10^6^ cells/mL had 82% positive predictive value and 78% negative predictive value for death, with a relative risk of death of 9.33 (*p* = 0.009, Fig. 4 and Supplemental Table), and was independent of age, P:F ratio at enrollment, or requirement for vasopressors. This difference in survival by fibrocyte threshold was present at both day 28 (*p* = 0.028) and at the conclusion of the follow-up period (*p =* 0.009). This threshold concentration also identified ARDS patients at higher risk of prolonged mechanical ventilation and prolonged ICU stay (Supplemental Fig. 5).

## Discussion

Three lines of evidence support a role for fibrosis in ARDS: First, lung histology in surgical lung biopsies and autopsy series show evidence of fibrosis in 20–55% of patients (Papazian et al. 2007; Thille et al. 2013). Second, high-resolution chest CT scans demonstrate reticulation in 87% of patients by 2 weeks after onset of illness, which correlate with restrictive pulmonary function tests up to 180 days after the illness (Burnham et al. 2014) that persist for up to 5 years (Wilcox et al. 2013). Third, biomarkers of fibrogenesis, including N-terminal pro-peptide of type III collagen, C-terminal pro-peptide of type I collagen, TGF-β, and alveolar fibroblasts and fibrocytes are detected in the bronchoalveolar fluid as early as 24 h after the onset of ARDS and correlate with mortality, as reviewed in (Wang et al. 2019; Capelozzi et al. 2017). Surprisingly, lung fibrosis has become more, not less, prevalent during the low-tidal volume ventilation era as compared to previously (Thille et al. 2013), possibly due to a survivorship bias. Given the correlation between fibrogenesis and mortality in ARDS (Marshall et al. 2000; Quesnel et al. 2012; Chesnutt et al. 1997; Clark et al. 1995), early detection of this mechanism may be clinically important.

The study of diagnostic and prognostic biomarkers of ARDS has been an area of active investigation for three decades, but few peripheral blood biomarkers of fibrosis have been identified to date (Capelozzi et al. 2017; Terpstra et al. 2014). Fibrocytes have been shown to expand in the blood in the context of both physiologic wound repair and several diseases associated with pathologic fibrogenesis in diverse diseases (Trimble et al. 2014; Shipe et al. 2016; Field et al. 2012; Moeller et al. 2009; Just et al. 2017; Andersson-Sjöland et al. 2009; Andersson-Sjöland et al. 2008). Given that the presence of fibrocytes in the bronchoalveolar lavage fluid in ARDS correlates with poor outcomes (Quesnel et al. 2012; Quesnel et al. 2010), we sought to determine whether circulating concentration of fibrocytes may prove relevant as a biomarker.

In the context of the animal model of acute lung injury caused by an experimental hypervirulent strain of *Klebsiella pneumoniae*, we found a time-dependent increase in blood fibrocytes, followed by the lungs, which correlated with increasing concentrations of lung soluble collagen, analogous to prior reports in models of bleomycin- and fluorescein-induced lung fibrosis and sickle cell lung disease (Field et al. 2012; Mehrad et al. 2009; Moore et al. 2005; Phillips et al. 2004). We similarly found an expansion of circulating fibrocytes in patients with pneumonia and ARDS, which disproportionately expressed myofibroblast markers and expressed phosphorylated SMAD-2/3, evidence of TGF-β receptor signaling. These findings are reminiscent of reports in fibrotic lung diseases, COPD, and asthma (Trimble et al. 2014; Shipe et al. 2016; Sun et al. 2016; Dupin et al. 2016), suggesting that expansion of activated fibrocytes in the blood and their recruitment to the lungs may be indicative of a general program of fibrogenesis that is a feature of diverse forms of lung injury.

CXCR4 was the most prevalent fibrocyte chemokine receptor in the bone marrow, blood, and lung in the animal model, and in the blood in human subjects with ARDS (Figs. 1d, 2c, and Supplemental Fig. 1), and we noted a time-dependent progressive gradient of the CXCR4 ligand, CXCL12, between the three compartments during the course of lung injury in the animal model (Fig. 1e-f). Although these data are consistent with the hypothesis that the CXCR4-CXCL12 axis mediates recruitment of fibrocytes to the lungs during ARDS, several other chemokine (Moore et al. 2005; Moore et al. 2006; Ishida et al. 2017; Ishida et al. 2007) and non-chemokine (Wang et al. 2015; García-de-Alba et al. 2010; Katebi et al. 2008; Aono et al. 2014) systems can mediate fibrocyte migration, and these mechanisms may operate in parallel or in series.

In patients with ARDS who were studied longitudinally, we observed an unexpected pattern of intermittent but marked elevations of blood fibrocyte concentrations during the ICU stay. This contrasted with the animal model which displayed a monophasic increase and subsequent decline in the concentration of circulating fibrocytes (Figs. 2a and 3a). Interestingly, elevated percentage of circulating fibrocytes at a single time point has been correlated with increased mortality in patients with ARDS (Tai et al. 2018). The present work adds to this literature by showing that subjects with ARDS develop episodic marked elevations in circulating fibrocytes that were predictive of outcomes, similar to patients with fibrotic interstitial lung diseases (Thille et al. 2013; Moeller et al. 2009). There are few reports of serial measurements of circulating fibrocytes in human diseases in the literature, but we noted a similar pattern of intermittent elevations of circulating fibrocyte concentration in a genetic form of interstitial lung disease (Thille et al. 2013) albeit over a longer time-scale. We hypothesize that, in patients with ARDS, the intermittent elevations in blood fibrocytes may represent new episodes of injury acquired in the ICU, such as ventilator-associated lung injury or infections, that cumulatively contribute to poor outcomes.

We recognize several limitations in this study. First, this was an observational study, in which we did not specifically study the mechanism by which fibrocytes contribute to the evolution of ARDS; as such, these data do not allow causal inferences about the role of fibrocytes in pathogenesis of lung injury. Second, even though the mortality rate was low, the results of our animal studies at later time points could be confounded by a survivorship bias. Third, the results from our clinical cohort, although notable for representing longitudinal biomarker data in a representative population of patients with lung injury, represents data obtained from a small, single-center study, which awaits external validation. Related to this point, our study was not adequately powered to determine whether elevated fibrocyte concentrations at early time points have a different implication from elevations at later points. Finally, we did not identify the specific cause of episodic fibrocyte elevation in the circulation of patients with ARDS. Thus, while these events correlated with mortality, their etiology remains speculative.

## Conclusions

In summary, we report the expansion of fibrocytes in the bone marrow, blood, and lung of animals with experimental lung injury that coincided with lung fibrogenesis, and show a similar pattern in blood fibrocytes in patients with lung injury, the extent of which was predictive of outcomes. Based on these results, we propose a model wherein lung fibrogenesis is detectable by serial monitoring of blood fibrocytes in ICU patients, and therefore may help predict outcomes in these critically ill patients.

## Supplementary information

**Additional file 1: Figure S1.** Distribution of median percentage of fibrocytes in the bone marrow and peripheral blood of mice on day 3 after onset of acute lung injury caused by experimental *Klebsiella* pneumonia, expressing the chemokine receptors CXCR4, CCR2, and CCR7. **Figure S2.** Comparison of initial total fibrocytes (CD45+ Col1+) and activated fibrocytes staining for phosphorylated SMAD-2 or − 3 (CD45+ Col1+ pSMAD2/3+) or expressing alpha-smooth muscle actin (CD45+ Col1+ α-SMA+) in healthy human subjects, and subjects with pneumonia (PNA) or acute respiratory distress syndrome (ARDS). Each dot represents one subject; bold horizontal lines indicate the median, and light horizontal lines represent the 25th and 75th percentiles. **Figure S3.** Distribution of the median percentage of circulating fibrocyte expressing the indicated chemokine receptors on the day of peak activated fibrocyte concentration in patients with ARDS. **Figure S4.** Comparison of plasma levels CXCL12, latent and active TGF-β, C-terminal propeptide of collagen I (PCICP), N-terminal propeptide of collagen I (PCINP), and N-terminal propeptide of collagen III (PCIIINP) in healthy human subjects and subjects with pneumonia or ARDS on the day of peak circulating activated fibrocyte concentration. **Figure S5:** Kaplan-Meier analysis of subjects with ARDS, separated by peak circulating activated fibrocyte concentration threshold value of 4.8 × 10^6^ cells/mL with analysis comparing total time on mechanical ventilation (A) or ICU length of stay (B). **Table S1.** Logistic regression models used to predict death.

## Data Availability

The datasets used and/or analyzed during the current study are available from the corresponding author on reasonable request.

## Abbreviations

ARDS: acute respiratory distress syndrome
ICU: intensive care unit
BA: bronchoalveolar lavage
