# Supplementary material for: Circulating fibrocytes traffic to the lung in murine acute lung injury and predict outcomes in human acute respiratory distress syndrome: a pilot study
Source: Mol Med. 2020 May 27;26:52. doi: 10.1186/s10020-020-00176-0 (PMC7251319; doi:10.1186/s10020-020-00176-0)
Supplement: Supplementary file 1 — Additional file 1: Figure S1. Distribution of median percentage of fibrocytes in the bone marrow and peripheral blood of mice on day 3 after onset of acute lung injury caused by experimental Klebsiella pneumonia, expressing the chemokine receptors CXCR4, CCR2, and CCR7. Figure S2. Comparison of initial total fibrocytes (CD45+ Col1+) and activated fibrocytes staining for phosphorylated SMAD-2 or − 3 (CD45+ Col1+ pSMAD2/3+) or expressing alpha-smooth muscle actin (CD45+ Col1+ α-SMA+) in healthy human subjects, and subjects with pneumonia (PNA) or acute respiratory distress syndrome (ARDS). Each dot represents one subject; bold horizontal lines indicate the median, and light horizontal lines represent the 25th and 75th percentiles. Figure S3. Distribution of the median percentage of circulating fibrocyte expressing the indicated chemokine receptors on the day of peak activated fibrocyte concentration in patients with ARDS. Figure S4. Comparison of plasma levels CXCL12, latent and active TGF-β, C-terminal propeptide of collagen I (PCICP), N-terminal propeptide of collagen I (PCINP), and N-terminal propeptide of collagen III (PCIIINP) in healthy human subjects and subjects with pneumonia or ARDS on the day of peak circulating activated fibrocyte concentration. Figure S5: Kaplan-Meier analysis of subjects with ARDS, separated by peak circulating activated fibrocyte concentration threshold value of 4.8 × 10^6^ cells/mL with analysis comparing total time on mechanical ventilation (A) or ICU length of stay (B). Table S1. Logistic regression models used to predict death. [file 10020_2020_176_MOESM1_ESM.docx]

**SUPPLEMENTAL DIGITAL CONTENT**

**Circulating fibrocytes traffic to the lung in murine acute lung injury and predict outcomes in human acute respiratory distress syndrome**

Christine M. Lin, MD; Abdullah Alrbiaan, MD; John Odackal, MD; Zhimin Zhang, MD;

Yogesh Scindia, PhD; Sun-Sang J. Sung, PhD; Marie D. Burdick, BS; Borna Mehrad, MD


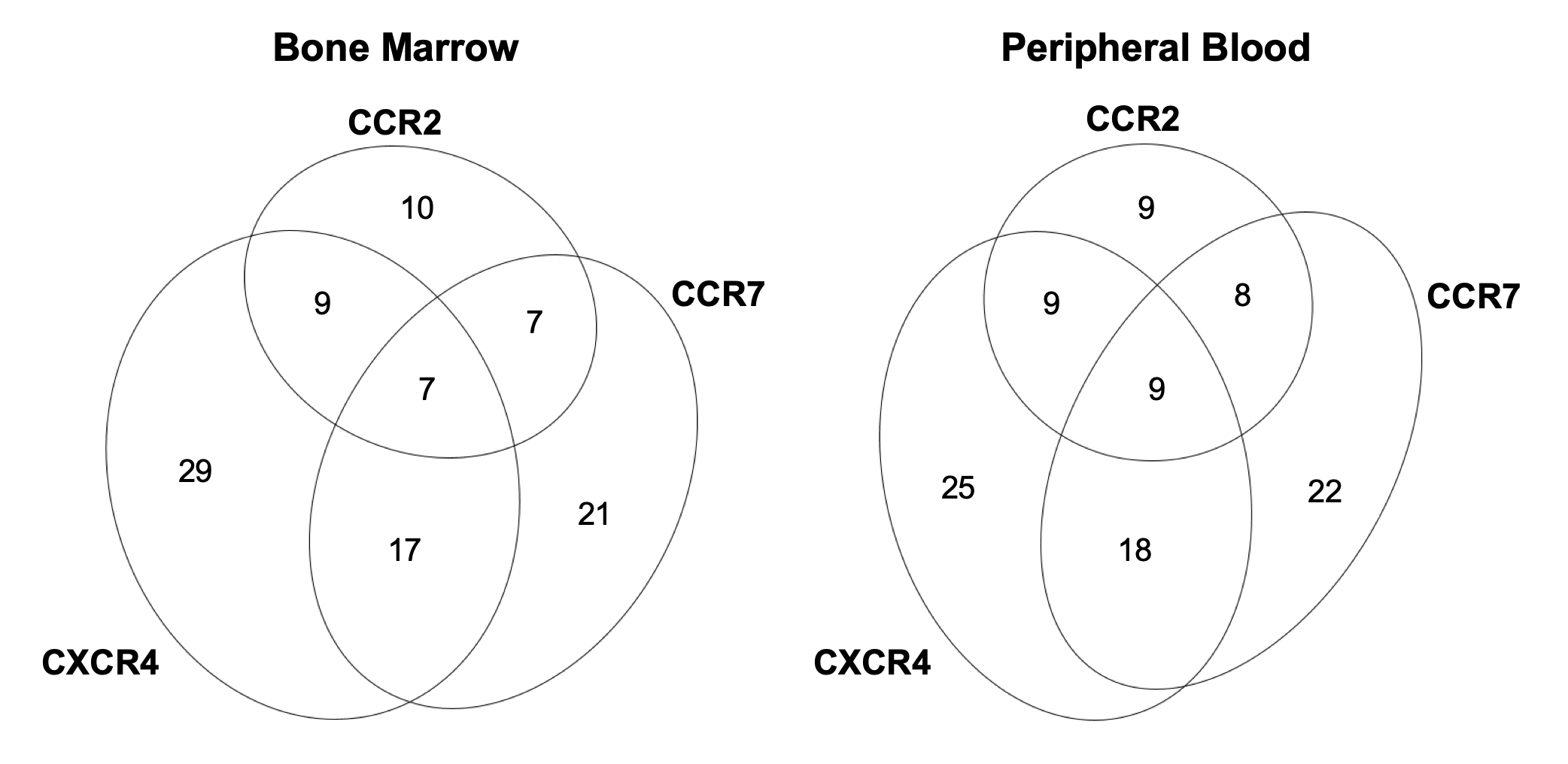


**Supplemental Figure 1:** Distribution of median percentage of fibrocytes in the bone marrow and peripheral blood of mice on day 3 after onset of acute lung injury caused by experimental *Klebsiella* pneumonia, expressing the chemokine receptors CXCR4, CCR2, and CCR7.

**Supplemental Figure 2:** Comparison of initial total fibrocytes (CD45+ Col1+) and activated fibrocytes staining for phosphorylated SMAD-2 or -3 (CD45+ Col1+ pSMAD2/3+) or expressing alpha-smooth muscle actin (CD45+ Col1+ α-SMA+) in healthy human subjects, and subjects with pneumonia (PNA) or acute respiratory distress syndrome (ARDS). Each dot represents one subject; bold horizontal lines indicate the median, and light horizontal lines represent the 25th and 75th percentiles. Red dots indicate patients that required mechanical ventilation; open dots indicate patients that required vasopressors. *, *p*<0.05; **, *p*<0.01; ***, *p*<0.001 by Kruskal-Wallis test.

**Supplemental Figure 3:** Distribution of the median percentage of circulating fibrocyte expressing the indicated chemokine receptors on the day of peak activated fibrocyte concentration in patients with ARDS.

**
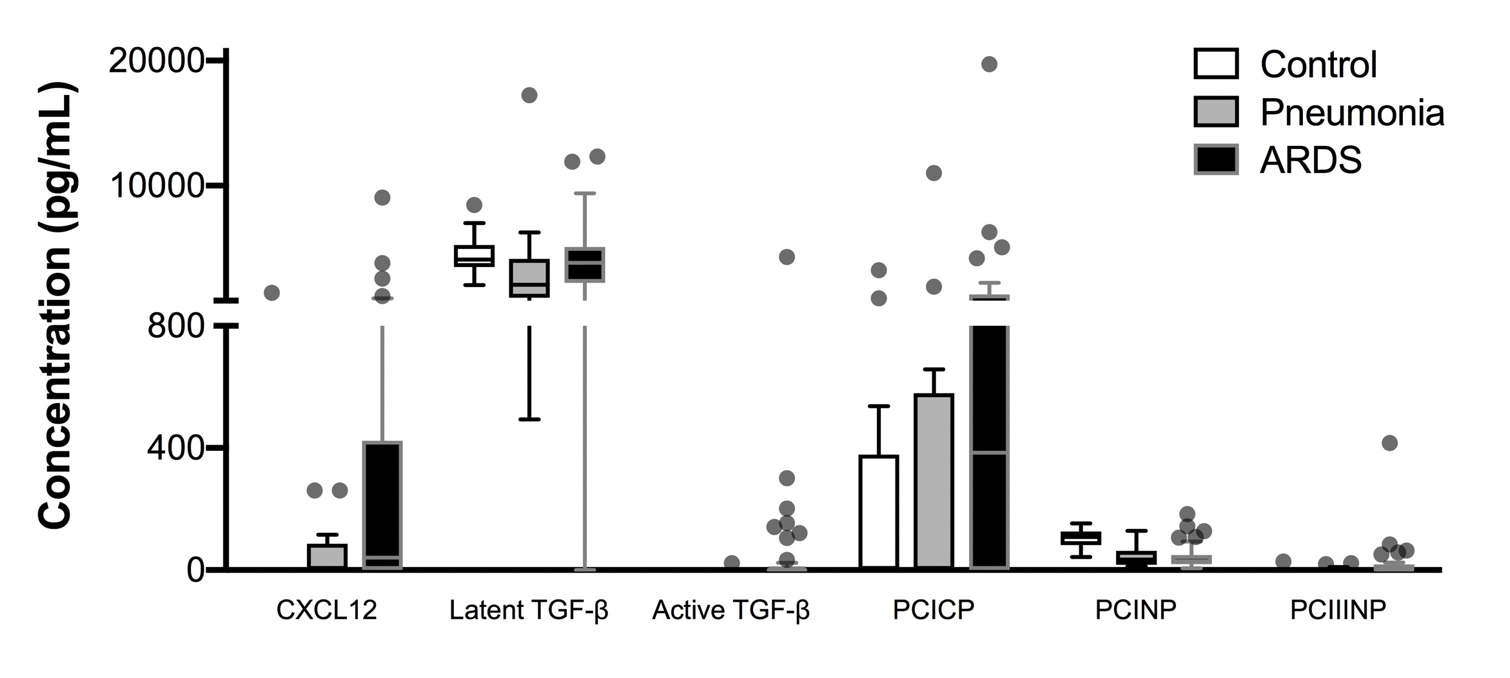
**

**Supplemental Figure 4:** Comparison of plasma levels CXCL12, latent and active TGF-β, C-terminal propeptide of collagen I (PCICP), N-terminal propeptide of collagen I (PCINP), and N-terminal propeptide of collagen III (PCIIINP) in healthy human subjects and subjects with pneumonia or ARDS on the day of peak circulating activated fibrocyte concentration. No statistically significant difference in any biomarker was discernable between groups by Kruskal-Wallis test.

**Supplemental Figure 5:** Kaplan-Meier analysis of subjects with ARDS, separated by peak circulating activated fibrocyte concentration threshold value of 4.8 x 10^6^ cells/mL with analysis comparing total time on mechanical ventilation (A) or ICU length of stay (B). Of the 42 subjects with ARDS, 9 subjects had values above and 33 subjects had values below the threshold, *, *p*<0.05.

| **Parameter** | | **Odds ratio estimate (95% CI)** | | | |
| --- | --- | --- | --- | --- | --- |
|  | Univariate model (n=42) | | Multivariate model 1 (n=42) | Multivariate model 2 (n=42) | Multivariate model 3 (n=42) |
| Fibrocyte concentration above threshold | 9.33  (1.63 – 53.6) | | 9.31  (1.62 – 53.5) | 9.33  (1.60 – 54.5) | 7.30  (1.20 – 44.6) |
| Age |  | | 1.00  (0.96 – 1.05) |  |  |
| P:F ratio |  | |  | 1.00  (0.98 – 1.01) |  |
| Vasopressor use |  | |  |  | 4.55  (0.79 – 26.3) |

**Supplemental Table:** Logistic regression to predict death.
